# Thai dance exercises benefited functional mobility and fall rates among community-dwelling older individuals

**DOI:** 10.1142/S1013702520500031

**Published:** 2019-12-16

**Authors:** Chonticha Kaewjoho, Lugkana Mato, Thiwabhorn Thaweewannakij, Saowanee Nakmareong, Supaporn Phadungkit, Chitanongk Gaogasigam, Sugalya Amatachaya

**Affiliations:** 1 School of Physical Therapy, Faculty of Associated Medical Sciences, Khon Kaen University, Khon Kaen, Thailand; 2 Improvement of Physical Performance and Quality of Life (IPQ), Research Group, Khon Kaen University, Khon Kaen, Thailand; 3 Department of Physical Therapy, Faculty of Allied Health Sciences, Chulalongkorn University, Bangkok, Thailand; samata@kku.ac.th

**Keywords:** Older adult, fall, balance, walking, cultural dance

## Abstract

**Background:**

With dramatic increase in the number of older individuals, special efforts have been made to promote the levels of independence and reduce fall rates among these individuals.

**Objective:**

To investigate the effects of Thai dance exercises over 6 weeks on functional mobility and fall rates in community-dwelling older individuals.

**Methods:**

Sixty-one community-dwelling older adults were interviewed and assessed for their demographics and fall data during 6 months prior to participation in the study. Then they completed the quasi-experimental Thai dance exercise program for 50 minutes/day, 3 days/week over 6 weeks. Their functional mobility relating to levels of independence and safety were assessed prior to training, at 3-week and 6-week training. After completing the program at 6 weeks, participants were prospectively monitored for fall data over 6 months.

**Results:**

Participants improved their functional mobility significantly after 3- and 6-week training (p<0.01). The number of faller individuals obviously decreased from 35% (n=21) prior to training to only 8% (n=5) after training (p<0.01).

**Conclusion:**

The current findings further extend benefits of Thai dance as an alternative musical exercise program to promote levels of independence and safety among community-dwelling older adults.

## Introduction

Advancing age commonly accompanies many system declines that affect several contributors to independence and risk of falls among older individuals, including a safe and efficient ambulatory status, good static and dynamic balance, adequate lower extremity muscle strength, and good functional endurance.^1,2^ Therefore, special efforts have been made to promote levels of independence and reduce fall rates among these individuals, particularly in the current era, whereby the number of older adults has obviously increased.

Many exercise programs have been reported for their effectiveness in promoting the physical performance of older individuals.^1,3,4^ Among existing programs, musical and dance exercise programs have been reported to enhance recruiting and retaining of older individuals in the exercise programs.^5^ However, the existing reports on cultural dance programs — Brazilian, Turkish and Greek dances — have their own characteristics and varying options based on the country of origin that are suitable for their populations.^6,7^ Thus, they may be difficult to be applied for Thai older individuals, i.e., need special and long training duration to be familiarized in the training programs.

By contrast, Thai dance exercise is well known and widely used by Thai people. The program is characterized by smooth, gentle, and coordinated movements involving the whole body^8^ that might be particularly challenging for the important contributors to the independence, community participation, and safety of older individuals such as balance and walking ability, lower limb muscle strength, and functional endurance.^2,5^ However, there are few studies reporting benefits of Thai dance exercises, and only in female individuals without consideration of all variables needed for being independent, along with fall rates of the participants.^9,10^ Further exploration on the effects of Thai dance exercises in both male and female individuals on variables necessary for being independent and fall rates would extend the clinical implication of the program for older adults. Therefore, this study compared the effects of Thai dance exercises over 3 and 6 weeks on the functional mobility necessary for being independent, including the timed up and go test (TUG), five times sit-to-stand test (FTSST), 10-meter walk test (10 MWT), and 6-minute walk test (6 MinWT)^2,5^ and fall rates among community-dwelling older individuals.

## Materials and Methods

### Study design and population

This quasi-experimental study was conducted in community-dwelling older adults, aged 65 years and over, from several rural communities in Thailand, during November 2016 and September 2018. The eligible participants had to walk independently over at least 10 m without any assistive devices, and had not participated in a regular exercise program prior to being involved in the study. Older individuals who presented any signs and symptoms that might affect walking and the ability to participate in this study, such as unstable medical conditions, inflammation in the joints of the lower extremities (with a pain scale of more than 5 out of 10 on a visual analog scale), and having sequelae of neurological deficits, were excluded from the study.^2,11,12^ The study protocol was approved by the local ethics committee (HE 602099) and eligible individuals signed a written informed consent before taking part in the study (clinical trial registration number NCT02919514).

### Sample size calculation

The sample size was estimated from data of a pilot study (n=18) for the primary outcome, the 10 MWT, with the effect size of 0.09 m/s, power of test at 80%, alpha level of 0.05, and a dropout rate of 20%. The findings indicated that the study required at least 55 participants.

### Research protocols

Older adults who agreed to be involved in the study were screened and assessed for their eligibility according to the study criteria. Then, the eligible participants were interviewed and assessed for their demographics, including age, gender, body mass index, health status, and fall data over the previous six months prior to participation in the study, with data confirmation from their relatives and related events (if any), i.e., the date, time, place, circumstances, consequences, and treatment required. On the next day, participants were assessed for functional outcomes of the study. Then, they were trained using standard protocols of traditional Thai dance following video demonstration, and they subsequently became involved in a traditional Thai dance exercise program in their communities. Details of the training and testing protocols are as follows.

#### Thai dance exercise training

**Fig. 1.**
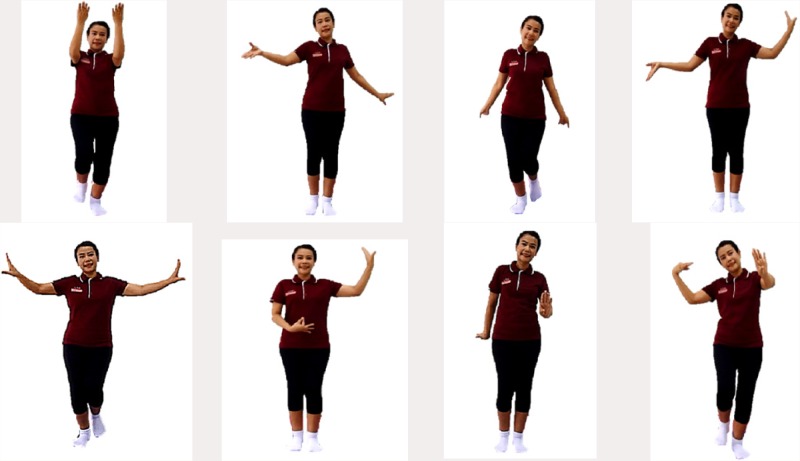
Examples of Thai dance exercise maneuvers.

The program consisted of a warm-up session for 10 min, Thai dance exercises for 30 min, and a cool-down and muscle-stretching session for 10 min. The Thai dance exercises were performed with a video demonstration of the standard traditional Thai dance using eight songs, including the *Ngam sang duan, Chaw Thai, Ram ma si ma ram, Dong jan wan pen, Dok mai kong chat, Ying Thai jai ngam, Dong jan kwan fa*, and *Boo cha nak rop* songs. These songs required the participants to move and rotate their bodies while moving their arms upward, downward, and sideward alternately (Fig. 1). During these movements, both legs needed to step forward and backward with slight flexion of the knees on either a single or double limb support period according to the rhythm of the songs.^9^ Participants were able to take a period of rest during the training, as needed. However, after being involved in the program, they were encouraged to increase training time or minimize resting periods, as they could. The participants were trained for 50 min/day, 3 days a week for 6 weeks; thus, there were 18 sessions in total. Data of participants who participated in the training program for less than 15 sessions (80% of all training sessions) were excluded from the data analysis to clearly present the benefit of training over six weeks.^9^

#### Outcome measures

Participants were assessed for their functional mobility necessary for being independent, including the TUG, FTSST, 10 MWT, and 6 MinWT^2,5^ in a random sequence. Prior to the assessments, a group of three raters were trained for the testing protocols and standard commands were used for the tests. Then they practiced using the tests in 10 pilot older individuals, and the data showed that these rates had excellent inter-rater reliability (intraclass correlation coefficients [ICCs]=0.88–0.99). Subsequently, these raters were measured in the outcomes of the study three times, including prior to training (pre-test), after 3 weeks of training (intermediate test), and after 6 weeks of training (post-test). Then, participants were monthly monitored for fall data over 6 months using a fall diary and telephone interview. The details of each assessment are as follows.

*10-meter walk test*: The 10 MWT is a valid and reliable test (ICC=0.88–0.97), and its outcomes reflect the overall quality of gait, and ambulatory and health status of older individuals.^13-17^ The participants walked at a comfortable and fastest speed along a 10 m walkway, and the time used over the 4 m in the middle of the walkway was recorded to minimize acceleration and deceleration effects.^12-14^ Then, the findings were converted to a walking speed in meters/second (m/s).

*Timed up and go test*: The TUG is an excellent reliability test (ICC=0.97–0.99) that is widely used to measure the dynamic balance control, mobility and fall risk of older adults.^18,19^ The participants were instructed to rise from an armrest chair, walk around a traffic cone that was placed 3 m ahead of the front edge of the chair, and return to sit down on the chair in the fastest and safe manner. The test recorded the time from the command “go” until the participant’s back was against the backrest of the chair.^2,12,19^

*Five times sit-to-stand test*: The FTSST is a valid and excellent reliability test (ICC=0.97–0.99) where the outcomes reflect functional lower extremity muscle strength and dynamic balance control while changing postures from sitting to standing.^20-23^ Participants performed five repetitive chair-rise cycles at the fastest speed and in a safe manner without using their arms. The test records the time in seconds from the command “go” until the participant’s back touches the backrest of the chair after the fifth repetition.^2,12,22^

*6-minute walk test*: The 6 MinWT is an excellent reliability test (ICC=0.95) that is commonly used to represent functional endurance in community-dwelling older individuals.^24^ The test records the longest distance walked along a rectangular walkway (96 m) in 6 min. Every minute during the test, participants were informed of the time remaining and encouraged to continue walking as soon as they could. Then, the total distance covered after 6 min was recorded.^12,25^

The 6 MinWT was performed over one trial, and the 10 MWT, TUG, and FTSST were assessed over three trials, where the average findings were used for data analysis. During the tests, an assessor was beside or walked alongside the participants without interruption to ensure the participants’ safety and the accuracy of the outcomes. The participants wore a proper size of sandal sport shoes that were prepared by the researchers, and they were given a practice session so they could familiarize themselves with the shoes. Participants were able to take a period of rest during the study and the tests as needed.

*Fall surveillance*: After completing the program over six weeks, participants received a fall diary to record fall data and related events daily over six months. A researcher phoned them every month to gather the fall data of the month. Each fall was confirmed by related events (including the date, time, place, circumstances, and consequences of the fall) and by their caregivers or relatives to promote the accuracy of the interviewed data. A fall was defined as “an unintentional event that resulted in a person coming to rest on the ground from an upright standing or walking activity as a result of neither a major intrinsic event (stroke or syncope) nor an extrinsic cause”.^2,26^

### Statistical analysis

Descriptive statistics were used to describe the demographics of the participants and findings of the study. The analysis of variance with repeated measures (repeated measures ANOVA) was used to analyze the different findings among the three measurement times of the participants. The Chi-square test was used to compare fall data during 6 months before and after training. The level of significant difference was set at p-value of <0.05.

## Results

### Participants

**Fig. 2.**
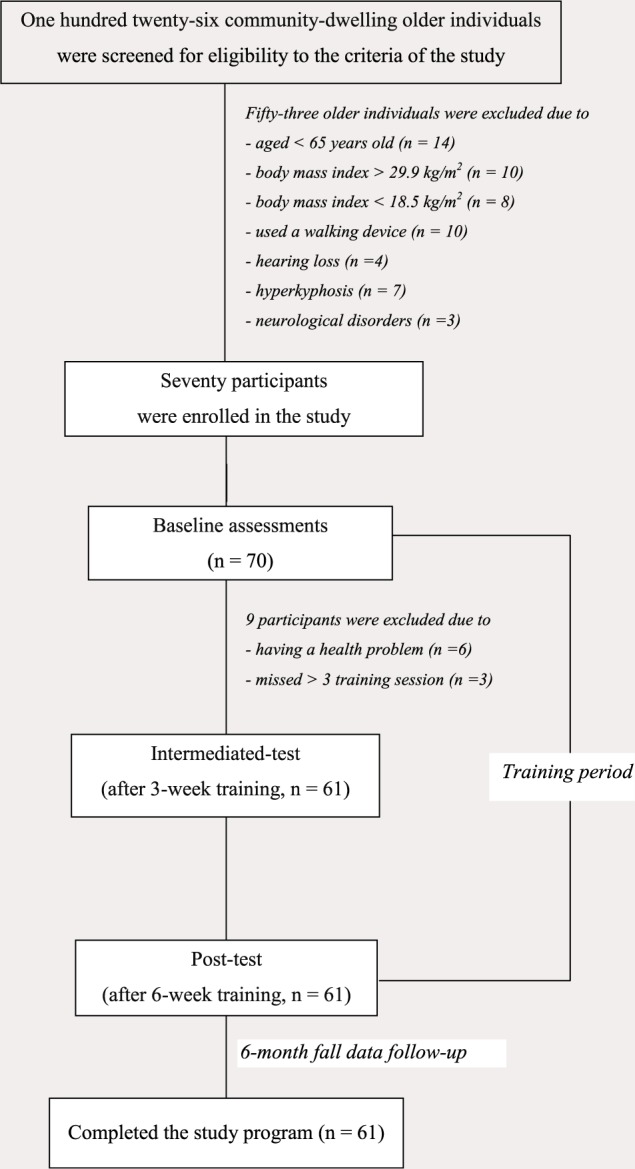
Participation flowchart.

Sixty-one participants, with an average age of 73 years and a normal body mass index, completed the study program (Fig. 2). Most were female (n=41), and 21 participants (35%) experienced at least one fall during 6 months prior to participation in the study, where most (n=20) had a single fall and one participant reported 2 falls (Table 1). The demographics between those who fell and did not fall showed no significant differences (p>0.05). Other baseline demographics are presented in Table 1.

**Table 1.** Baseline demographics and fall data of the participants (N=61).

Variable	Baseline data^c^	Post-training^d^
Age^a^ (years)	72.9 ± 5.7	
Weight^a^ (kg)	57.1 ± 7.7	
Height^a^ (cm)	155.1 ± 7.2	
Body mass index^a^ (kg/m^2^)	23.7 ± 2.9	
Number of medications^b^
Less than 4 types	51 (84)	
At least 4 types	10 (16)
Gender^b^
Female	41 (67)	
Male	20 (33)
Fall data${}^{\text{b}*}$	
No fall	40 (65)	56 (92)
Single fall	20 (33)	4 (6)
Multiple falls	1 (2)	1 (2)

*Notes*: ^a^The data are presented using mean ± standard deviation (95% confidence intervals), ^b^these variables were presented using number (%), ^c^the fall data were interviewed retrospectively over 6 months, ^d^the fall data were prospectively gathered over 6 months, ^∗^Chi-square test indicated significant difference with p-value <0.01.

### Outcomes of the study

After training, the participants showed significant improvements in all functional outcomes at week 3 and week 6 (p<0.01). The improvement was particularly demonstrated for the FTSST where the outcomes after the training differed significantly from both the pre-test and intermediate-test (p<0.01; Table 2). Furthermore, the number of those who fell was significantly reduced as compared to the data prior to training (p<0.01), where only five participants (8%) experienced falls during the 6-month prospective follow-up period (4 participants reported a single fall, where 1 of them was the same person who fell prior to training, and another one participant experienced 2 falls, Table 1).

**Table 2.** Functional mobility of the subjects at baseline, week 3, and week 6 (N=61).

Variable	Baseline (week 0)	Intermediate-test (weeks 3)	Post-test (weeks 6)
Ten meter walk test (m/s)			
Preferred speed	1.12±0.16 (1.08–1.16)	1.20±0.15 (1.17–1.24)^b^**	1.24±0.16 (1.20–1.28)^b^**
Fastest speed	1.38±0.19 (1.32–1.42)	1.45±0.22 (1.39–1.50)^b^**	1.43±0.19 (1.38–1.48)^b^*
Timed up and go test (s)	10.29±1.70 (9.86–10.73)	9.30±1.23 (8.99–9.63)^b^**	9.08±1.10 (8.80–9.36)^b^**
Five times sit-to-stand test (s)	13.52±2.62 (12.85–14.19)	11.28±2.48 (10.64–11.92)^b^**	10.16±1.94 (9.67–10.66)^^b^**,^I^**^
Six minute walk test (m)	332.2±48.8 (319.7–344.7)	349.9±47.7 (337.6–362.1)^b^**	354.7±46.6 (340.1–363.9)^b^**

*Notes*: The data are presented using mean ± standard deviation (95% confidence intervals). ^∗^Indicates the level of significant difference with the p-value <0.05, ${}^{**}p$-value <0.01. Superscripts indicate the measurement time with significant differences from the indicated period where ${}^{\text{b}}=$ baseline, and ${}^{\text{I}}=$ intermediate-test.

## Discussion

Thai dance is a part of the traditional art and culture that is familiar to Thai people. However, little evidence supporting the benefit of Thai dance exercises in only female individuals was available without the consideration of fall data of the participants.^9,10^ Thus, this study further assessed the effects of Thai dance exercises over 6 weeks on the functional mobility necessary for being independent, including the TUG, FTSST, 10 MWT and 6 MinWT, and fall rates over 6 months among community-dwelling older individuals. The findings indicated that participants improved all functional outcomes significantly since 3 weeks of training (p<0.01), and their functional mobility at 6 weeks showed further improvement, but not significantly different from the data at 3 weeks, except the FTSST (p<0.01, Table 2). Moreover, the number of faller individuals was obviously reduced (from 21 participants [35%] prior to training to only five participants [8%] after training, p<0.01).

The significant improvement after training may relate to characteristics of the Thai dance program that required participants to step forward and backward repeatedly while raising and lowering the body over the extended and bending of a single and double limb support period of the lower extremities (Fig. 1).^8^ The moderate rhythms of the songs also provided auditory cues to guide the participants to maintain their movement speed for over 30 min/day. Although some participants took a period of rest when they first became involved in the training program, they were encouraged to increase their participation duration to 30 min continuously as they could. Such training programs attributed both psychological and physical effects and are particularly challenging for important contributors to be independent in their daily living, such as lower limb muscle strength, balance control, walking ability, and functional endurance.^21,25^ Therefore, the participants showed significant improvement in all functional outcomes of the study (p<0.01, Table 2). However, the intermediate assessments at 3 weeks further suggested that the benefit of Thai dance exercise was demonstrated within a short period after training. The changes of these tests were also greater than the levels of clinical significance, i.e., greater than 0.05 m/s for the 10 MWT,^27^>9% changes for the TUG,^28^ and 20 m for the 6 MinWT.^27^ Therefore, the findings further extend the benefit of Thai dance exercise program over 3 and 6 weeks on functional ability of older adults necessary for being independent.

Of all the functional measures, an improvement was obviously found for the FTSST, where the improvement after 6 weeks was significantly different from their baseline ability (3.36 s) and intermediate assessments ( p<0.01, Table 2). This improvement was greater than that used to determine clinical significance (2.3 s).^29^ The findings may reflect effects of the training program that required the participants to bend the knees always while moving their arms and body forward, backward, and sideward. Repetitive practice in such tasks may particularly challenge the lower limb muscle strength and dynamic balance ability that is necessary to complete the FTSST.^20,30^ The improvement of FTSST is important as it is widely used to predict lower limb disability and independent living among older individuals.^20,22,31,32^

The improvement in the outcomes of these tests is also important for fall prevention among older individuals.^2,19,21,25^ Thus, the number of participants who fell was significantly reduced from 21 participants to only 5 participants (Table 1). Nonetheless, the fall data from retrospective and prospective follow-up may contain some sources of bias, e.g., recalling bias and Hawthorne effects that may affect data comparisons in the findings. In addition, fall events can occur due to various extrinsic causes, such as environmental hazards,^2,19^ that need to be taken into consideration for data interpretation.

The current findings were coherent with other cultural dances, such as Greek, Brazilian, and Turkish on the improvement of balance and physical ability.^6,7,33^ However, the researchers^6^ recommend that these dances are specific to the country of origin, and thereby may not allow generalization. The current findings further extend the benefit of Thai dance exercise for ability of being independent and on fall rates in participants who had good adherence to the Thai dance program (at least 80% of the total session (15 sessions).^34,35^ The intermediate assessments also suggest the benefit over a short training duration (3 weeks). Thus, the findings further confirm the use of Thai dance exercise as an alternative training program familiar to Thai community-dwelling older individuals. However, the findings were derived from a quasi-experimental design without a control group to ensure time-frame effects due to daily activities. Nevertheless, a previous report using a similar program found no significant difference in the control group that did not receive any additional program over 6 weeks.^9^ Moreover, with a single group study, the assessors were aware of the training program received by participants. However, the researchers attempted to minimize assessor-bias by using a group of excellent reliability raters (ICCs=0.88–0.99). In addition, the findings did not analyze the data on recruiting and retaining rates, and did not measure the outcomes over a retention period. Therefore, a further study should apply a randomized controlled trial with the assessments of recruiting and retaining rates and a measurement during a retention period to thoroughly confirm the effects of Thai dance exercises.

## Conclusion

Thai dance exercise program improved functional mobility of the participants after 3- and 6-week of training, as well as reduce the fall rates of older individuals. Hence, the present findings further confirm the use of a Thai dance exercise program, which is familiar to Thai individuals, as an alternative strategy to promote independence and safety among community-dwelling older adults.

## Conflict of Interests

The authors declared no potential conflict of interests.

## Funding/Support

This study was funded by National Research Council Thailand (6100119), and the Improvement of Physical Performance and Quality of Life (IPQ) Research Group (IPQ/SC-014), Khon Kaen University, Khon Kaen, Thailand.

## Author Contributions

All authors were responsible for research conception and design, critical revision of the article for important intellectual content, provision of study materials or patients. CK was additionally involved in the data acquisition, statistical analysis, and drafting of the manuscript. SA was also responsible for project management, funding application, and finalizing the manuscript.
